# Recent Advances in the Study of Alphaherpesvirus Latency and Reactivation: Novel Guidance for the Design of Herpesvirus Live Vector Vaccines

**DOI:** 10.3390/pathogens13090779

**Published:** 2024-09-10

**Authors:** Shinuo Cao, Mo Zhou, Shengwei Ji, Dongxue Ma, Shanyuan Zhu

**Affiliations:** 1 Jiangsu Key Laboratory for High-Tech Research and Development of Veterinary Biopharmaceuticals, Jiangsu Agri-Animal Husbandry Vocational College, Taizhou 225306, China; shinuo_cao@163.com (S.C.); zhoumo_wk@hotmail.com (M.Z.); 2 Department of Veterinary Medicine, Agriculture College of Yanbian University, Yanji 133000, China; jishengwei0903@hotmail.com

**Keywords:** alphaherpesviruses, neurons, latency, reactivation, live vector vaccine

## Abstract

Alphaherpesviruses, including herpes simplex virus type 1 (HSV-1), herpes simplex virus type 2 (HSV-2), and varicella-zoster virus (VZV), infect a diverse array of hosts, spanning both humans and animals. Alphaherpesviruses have developed a well-adapted relationship with their hosts through long-term evolution. Some alphaherpesviruses exhibit a typical neurotropic characteristic, which has garnered widespread attention and in-depth research. Virus latency involves the retention of viral genomes without producing infectious viruses. However, under stress, this can be reversed, resulting in lytic infection. Such reactivation events can lead to recurrent infections, manifesting as diseases like herpes labialis, genital herpes, and herpes zoster. Reactivation is a complex process influenced by both viral and host factors, and identifying how latency and reactivation work is vital to developing new antiviral therapies. Recent research highlights a complex interaction among the virus, neurons, and the immune system in regulating alphaherpesvirus latency and reactivation. Neurotropic alphaherpesviruses can breach host barriers to infect neurons, proliferate extensively within their cell bodies, and establish latent infections or spread further. Whether infecting neurons or spreading further, the virus undergoes transmission along axons or dendrites, making this process an indispensable part of the viral life cycle and a critical factor influencing the virus’s invasion of the nervous system. Research on the transmission process of neurotropic alphaherpesviruses within neurons can not only deepen our understanding of the virus but can also facilitate the targeted development of corresponding vaccines. This review concentrates on the relationship between the transmission, latency, and activation of alphaherpesviruses within neurons, summarizes recent advancements in the field, and discusses how these findings can inform the design of live virus vaccines for alphaherpesviruses.

## 1. Introduction

A number of alphaherpesviruses, including human herpesvirus 1 and 2 [1,2,3,4], bovine herpesvirus 1 (BHV-1) [5,6,7,8,9], varicella-zoster virus (VZV) [10,11], and pseudorabies virus (PRV), are able to affect their hosts in either a lytic or latent manner [12,13,14,15,16]. While their replication dynamics during the lytic cycle are well understood, there are nuances in their latent infection phase. For PRV and similar viruses, the latent infection phase primarily involves the long-term presence of the viral genome in the host without producing new viral progeny [12,14]. Stress can trigger these dormant viruses to resume their lytic activity, a process known as reactivation [3,14]. Alphaherpesviruses initially infect the skin or mucosal epithelium and subsequently enter neuronal axons, traveling retrogradely to the neuron cell bodies in ganglia, where they establish lifelong latency [3,17,18,19,20]. Periodically, these viruses reactivate and move anterogradely along neuronal axons back to mucosal or skin surfaces, where they replicate and cause epithelial lesions [21]. The anterograde transport of these viruses is crucial to their ability to exit neurons, spread within epithelia, and propagate new infections [22,23,24]. It has long been hoped that vaccines against widespread, and sometimes devastating, viruses could be developed by selectively removing retrograde delivery to the nervous system [25]. Decades of work have gone into various candidate alphaherpesvirus vaccines [26], and advances in the development of live-attenuated herpesvirus vaccines have concentrated on two principal approaches: broadly inhibiting virus propagation, or specifically hindering the virus’s ability to infect nervous cells while still allowing replication in nonneuronal cells. The latter strategy employs mutant viruses lacking specific envelope proteins that are essential for herpesviruses’ entry into neuronal cells [27,28,29]. Gaining a deeper understanding of the mechanisms underlying the neuronal transmission, latency, and activation of alphaherpesviruses is essential for designing innovative therapeutic approaches to manage their latency and could provide valuable insights for developing new live-attenuated herpesvirus vaccines.

## 2. Relationship between Latency, Reactivation, and Neural Transport of Alpha Herpesviruses

The traditional definition of latency refers to the persistence of a viral genome within tissue without a detectable infectious virus, detectable viral proteins, or detectable viral lytic transcripts. During this latent state, the virus remains dormant but retains the potential to be reactivated [30]. Latency is a stage during which the virus remains dormant within the host cells, evading the immune system’s active surveillance. Alphaherpesviruses reactivate from lifelong latency in their hosts. When they reactivate, they are able to spread within their hosts despite the presence of a potent immune response that includes neutralizing antibodies. This state is characterized by the minimal expression of viral proteins and nucleic acids, making the virus virtually undetectable. Reactivation occurs when a latent virus resumes active replication. During reactivation, the virus begins to produce new viral particles, which can lead to the re-emergence of symptoms and increase the likelihood of transmission to new hosts. The anterograde and retrograde transport of viral particles is essential for the latent infection and reactivation of alphaherpesviruses, as well as for the subsequent reinfection of tissues innervated by nerves and the efficient transmission of the virus between hosts [20,31,32,33].

**Figure 1:**
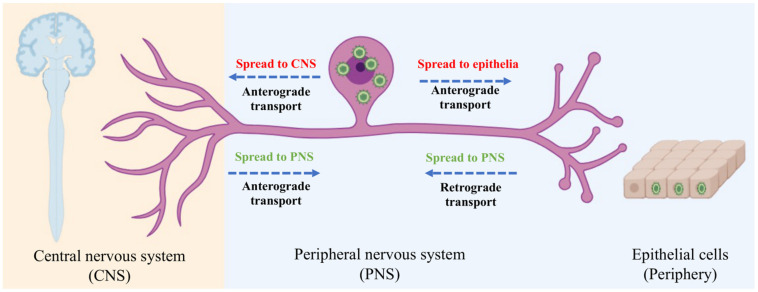
Directional spread of alphaherpesvirus entering the mammalian nervous system.

The spread of alphaherpesviruses within the nervous system includes long-distance trans-synaptic transmission between adjacent neurons [34,35,36,37]. Before invading the nervous system, alphaherpesviruses typically infect somatic cells, such as epithelial cells [38]. After replicating in these cells, the virus enters neurons by invading nerve endings distributed in the tissue. To further invade the host’s nervous system, viral particles undergo retrograde transport to the cell body upon entering nerve endings and establish lifelong latent infections in the cell bodies of sensory neurons [39,40]. However, establishing a latent infection in the natural host is based on a smaller amount of the virus invading the nervous system. If a large number of viral particles invade the host’s neurons, they will replicate in the cell body and undergo further transmission [41]. Latent infections can be reactivated when the host is under stress or in certain special conditions. Upon reactivation, newly synthesized viral particles undergo anterograde transport to the periphery via axonal sorting, often causing lesions in the host’s epithelial tissues (Figure 1) [42]. For instance, recurrent epithelial lesions are caused by HSV-1 and HSV-2 infections. Occasionally, reactivated viruses may spread to the central nervous system, which is significantly associated with the lethal outcomes of alphaherpesvirus infections, such as encephalitis caused by PRV infection in pigs [43,44].

## 3. Viral Proteins Involved in Anterograde of Viral Particles

The anterograde transport process of alphaherpesviruses is mainly divided into two phases: first, viral particles undergo retrograde transport to the cell body for replication, and then they are transported anterogradely along the axon back to the innervated epithelial tissue. Another process involves the viral particles replicating in the cell body and then being transported anterogradely along the axon or dendrites to higher-order neurons, eventually reaching more advanced neurons through trans-synaptic transmission [45]. Currently, it is believed that the anterograde transport of viral particles along the axon is mainly related to the interaction between viral-associated proteins and motor proteins, with motor proteins carrying viral particles along microtubules to complete the anterograde transport process.

Three viral membrane proteins (gE, gI, and US9) are essential for anterograde transmission within neural circuits in HSV and PRV [46,47]. Mutants lacking either the gE, gI, or US9 genes show significant differences in phenotype in vitro [48,49,50]. Mutants lacking the US9 gene completely lose the ability to transmit anterogradely across neurons [51,52,53]. In the absence of US9, viral particles can assemble in the cell body but cannot be sorted into the axon, yet the US9 gene deletion mutants are neuron-specific [48,52,54,55]. For PRV mutants lacking the gE and gI genes, anterograde transmission is still possible but is significantly reduced compared to the wild type [56]. gE and gI are both type I membrane proteins that form a heterodimer in the endoplasmic reticulum (ER). After leaving the ER, gE/gI primarily localizes to the Golgi apparatus, cytoplasmic vesicles, and the cell membrane [57,58]. Studies have demonstrated through biotin labeling of cell-surface proteins or the use of specific antibodies that gE/gI undergoes endocytosis from the cell membrane and accumulates in larger vesicles [59,60]. For PRV, the gE/gI complex is not essential for transneuronal spread in vivo. However, deletion of the cytoplasmic domain of gE or the full-length protein results in smaller plaques in nonneuronal cells, indicating its involvement in the spread between different cell types. In contrast, plaques formed by US9 deletion mutants are similar in size to those formed by wild-type PRV [1,61]. Overall, these results suggest that gE/gI and US9 have different roles in regulating anterograde transneuronal transmission.

## 4. Viral Proteins Involved in Retrograde Transport of Viral Particles

Although capsid transport in axons has a retrograde tendency, it remains bidirectional. This means that the virus undergoes not only retrograde transport but also brief periods of anterograde transport within the axon. However, anterograde transport constitutes only a very small part of the process by which the virus invades the cell body via the axon, thus the process is predominantly retrograde. Currently, the mechanisms of retrograde transport of viral particles in axons are still being explored and remain unclear. In this process, the virus uses the host’s dynein motor proteins to pull its nucleocapsid along the axon in retrograde transport, while the transport within the axon is regulated by host proteins [62].

Reports indicate that a small number of viral capsid and matrix proteins recruit molecular motors to facilitate retrograde transport in HSV and PRV, including UL36, UL37, and UL35 [29,63,64,65]. It was previously believed that UL36 was the main protein directly recruiting dynein, while UL37 or VP26 did not play a critical role in this process [66]. This is because viruses with UL36 mutations or antibodies injected against UL36 could block the delivery of capsid particles to the nucleus. In contrast, deletion or mutation of UL37 or VP26 did not seem to have the same effect [64]. Additionally, recent research has found that UL36 recruits dynein and dynein-activating proteins to facilitate the transport of viral particles during axonal transport. In this process, it interacts directly with the dynein intermediate chain and the subunits p150 and p50 of the dynein-activating protein, with the S region of UL36 playing a critical role [67]. The dynamic ubiquitination of the PRV UL36 protein significantly affects the virus’s ability to invade neurons. The dynamic changes between ubiquitination and deubiquitination are directly involved in the process of PRV breaching the host barrier to invade the nervous system from somatic cells and its transport along the axon within neurons [68]. The deubiquitination of UL36 assists the virus in breaching the host barrier to enter the nervous system, while the ubiquitination process drives the transport of viral particles along the axon, with the critical ubiquitination site being lysine 44 [65]. 

Recent studies have shown that mutating five amino acids in the R2 region of the UL37 protein results in the loss of retrograde transport capability, preventing the viral capsid from entering the cell body [69,70]. In this process, the mutant virus loses its retrograde tendency, increasing anterograde transport while decreasing retrograde transport, resulting in almost zero net displacement within the axon [29,70]. This research demonstrated the importance of UL37 in retrograde transport and suggests that it might function by regulating the interaction between viral capsid particles and motor proteins [29,71]. 

In addition, the attenuated PRV strain Bartha harbors several mutations affecting its virulence and neural circuit spread. This strain includes a genomic deletion that prevents anterograde spread, making it a widely used retrograde-restricted neural circuit tracer. Studies show that PRV Bartha’s retrograde spread is slower than that of wild-type PRV [72]. Researchers have utilized compartmented neuronal cultures to study the retrograde defect and uncover the genetic basis of the phenotype. PRV Bartha does not exhibit impaired retrograde axonal transport, but its transneuronal spread is reduced. Restoring the UL21 locus with wild-type sequences reinstated efficient transneuronal spread both in vitro and in vivo. Mutations in the Bartha UL21 gene likely cause defects in infectious particle production, delaying the spread to presynaptic neurons and infection amplification [73].

## 5. Other Viral Proteins Participating in Latency and Reactivation

The role of viral proteins in alphaherpesviruses’ neuroinvasion is a critical area of study, especially considering their roles in reactivation and latency [70]. Research spanning several decades has systematically explored how these proteins interact with host cells to facilitate both the initial infection and subsequent dormant phases [55,71,74]. Notably, thymidine kinase (TK) stands out as a vital virulence factor within this group of viruses [75,76]. Thymidine kinase’s function is particularly intriguing because it highlights the complex nature of viral behavior in different hosts and under varying biological conditions [77,78]. In a comprehensive study utilizing mouse models, it was found that strains of HSV-1 that are positive for TK expression produce significant titers in the central nervous system following corneal inoculation. Interestingly, nearly 80% of surviving mice established latency with these strains, demonstrating that this protein is crucial for both acute infection and latency establishment. Conversely, TK-negative strains present an entirely different scenario. These strains fail to invade the central nervous system or establish latency, indicating that TK expression is a key determinant of neuroinvasive capability and latent infection potential in mice [79]. A TK-negative mutant of HSV-1 with reduced neurovirulence also showed this differential response. Interestingly, while this mutant failed to establish latency in mice, it successfully established latency in rabbits, suggesting species-specific interactions that influence the outcome of infection [80]. This notion is further supported by findings from Coen and colleagues, who reported that while TK-negative mutants can establish latent infections in the trigeminal ganglia of mice, their ability to replicate acutely in these tissues is severely impaired. Moreover, these mutants did not reactivate from the ganglia when cocultured with permissive cells, emphasizing the critical role of TK in reactivation processes [76,81,82]. These studies collectively suggest that TK’s presence is not only pivotal for initiating the reactivation of HSV-1 but might also be dispensable for establishing latency. 

Alphaherpesviruses are widespread DNA viruses. These viruses are known for causing ulcerative mucosal lesions, infectious blindness, encephalitis, and severe neonatal illnesses. Their ability to initiate successful infections and maintain a persistent presence alongside the host’s immune defenses relies on their capacity to overcome antiviral responses. US11, an immune antagonist, is produced by both HSV-1 and HSV-2, as well as other primate viruses belonging to the *Simplexvirus* genus. US11 enhances viral production by inhibiting protein translation, critical for the virus’s survival and proliferation. The HSV-1 gK is essential for efficient replication and spread in the corneal epithelium and trigeminal ganglia neuroinvasion [83]. Audra et al. explored the effects of deleting US11 on the growth and pathogenicity of HSV-1 both in vitro and in vivo. Their study reinforced the significant role of US11 in both the disease process and the reactivation from latency, providing insights into how these medically significant viruses modulate immune responses [74]. 

Reactivation of latent alphaherpesviruses is triggered by various stimuli, including stress, immunosuppression, and neuronal activation [84]. The transition from latency to lytic replication involves the coordinated expression of viral genes, leading to the production of infectious virions. Key genes such as ICP0 and ICP4 initiate the lytic cycle and gene expression during reactivation, while early genes like ICP22 and ICP27 aid efficient viral replication and the transition from latency to the lytic phase. Late genes like gB, gD, TK, gE, and gI contribute to virion assembly, cell entry, and immune evasion during reactivation. Acute infection with animal alphaherpesviruses establishes a latency state in the trigeminal ganglia neurons [85]. 

## 6. Design Considerations for Alphaherpesvirus-Attenuated Vaccines

Utilizing alphaherpesviruses in live virus vector vaccines represents a promising avenue in vaccine development due to their ability to induce robust immune responses against target pathogens. One of the primary advantages of employing alphaherpesviruses as live virus vectors is their ability to establish lifelong latent infections in sensory neurons, providing long-lasting antigen presentation to the immune system. This characteristic makes them attractive candidates for vaccine vectors, as they can elicit both cellular and humoral immune responses against the inserted antigens.

Although this vaccine strain has good immunogenicity and protective efficacy, it can still invade neuronal cell bodies, posing a potential risk. The attenuated vaccine of PRV in pigs has good immunogenicity. However, the drawback is that inadequately attenuated vaccines may regain virulence and cause disease outbreaks. Additionally, the attenuated vaccine may establish latent infections or spread the virus to non-immunized pigs. Bartha-K61 was the first attenuated PRV vaccine to ever be developed, and it remains a standard vaccine used for eradicating Aujesky’s disease to this day [86]. It comprises an attenuated strain of the virulent PRV strain (Becker strain) that lacks the gE, gI, and US9 genes [87]. This strain exhibits significant differences in its ability to transport within neuronal axons compared to the Becker strain. While the Becker strain can transport bidirectionally within neuronal axons, the Bartha-K61 strain can only transport anterogradely [88]. The gE protein is essential for PRV to invade central nervous tissue from the retina, olfactory epithelial cells, and trigeminal ganglia, playing a decisive role in PRV’s neuroinvasiveness and transmission along nerves. The mechanism involves the formation of a dimer between gE and gI proteins, which indirectly promotes or stabilizes the interaction between the US9 protein and the motor protein KIF1A, facilitating the sorting of viral nucleocapsids and other proteins into neuronal axons for anterograde transport [49]. The gE, gI, and US9 genes are all associated with viral transport within neurons, indicating that the transport process in neurons affects the neurovirulence of the virus. 

Therefore, it is crucial to reduce the risk of establishing latent infections in the vaccine design process. We need to consider the effects of latency and reactivation on vaccine safety, immunogenicity, and efficacy during the process of designing a live virus vaccine for alphaherpesviruses. Ensuring that the live virus vector vaccine is safe for use in humans or target animals is paramount. Genetic modifications and attenuation strategies can enhance safety profiles. The vaccine should induce robust and long-lasting immune responses against key viral proteins involved in latency and reactivation. Clinical studies and animal models should be utilized to assess the vaccine’s efficacy in preventing both primary infection and reactivation events. 

## 7. Novel Design of Alphaherpesvirus-Attenuated Vaccine

Alphaherpesviruses are fascinating pathogens known for their ability to establish latent infections in the host nervous system and reactivate periodically. Understanding the genes and mechanisms underlying latency and reactivation is crucial for developing effective vaccines. Several strategies have been proposed to develop live-attenuated HSV vaccines, including compromising viral propagation broadly or targeting the virus’ ability to invade the nervous system while maintaining replication outside the nervous system. In the latter case, mutant viruses are generated that lack specific envelope proteins essential for the entry of HSV. The absence of the gE envelope protein prevents HSV from spreading to mouse dorsal root ganglia and provides protection against wild-type challenges [89,90,91].

Upon exposure to mucosal surfaces, neurotropic alphaherpesviruses spread to sensory and autonomic ganglia of the peripheral nervous system, serving as reservoirs for recurrent infections. The mechanism behind their nervous system invasion is unclear, but involves the pUL37 tegument protein, crucial for neuroinvasion. Mutations in this protein prevent HSV-1 and PRV from reaching the peripheral ganglia via retrograde axonal transport, reducing their virulence but not their peripheral replication [92]. Effective vaccines may utilize these findings by targeting the retrograde transport mechanism, potentially transforming approaches to preventing neurotropic viral diseases. The current live-attenuated PRV vaccines used in swine retain virulence in mice. However, the PRV pUL37 mutant, which lacks virulence in mice, still provides protection against challenges with wild-type PRV, making it an excellent indicator of vaccine effectiveness. This points to the potential of the pUL37 mutation as a foundation for developing vaccines aimed at various neuroinvasive herpesviruses. pUL37 mutants are incapable of infecting the peripheral nervous system, preventing latent infections but allowing replication in tissues peripheral to the nervous system. This effective replication in peripheral regions generates a strong immune response that protects against highly neuroinvasive strains of HSV-1 or PRV when exposed to the pUL37 mutant strain [93,94].

Pseudorabies virus (PRV), like other alphaherpesviruses, forms lifelong latent states in trigeminal ganglionic neurons (TG neurons). The latent viruses reactivate under stress, replicate in nasal mucosa nerve endings, and are shed in nasal and oral secretions, thereby spreading to naive animals. This cycle persists until the animal’s death or slaughter. Pavulraj et al. developed a PRV triple mutant (where TK, gG, and gE were replaced with chimeric genes) to be used as a live vaccination vector against classical swine fever virus (CSFV) and porcine circovirus 2b (PCV2b), and evaluated its latency reactivation compared with that of wild-type Becker virus [16]. Both strains established latency, but only the wild type replicated productively upon reactivation. The PRV triple mutant did not replicate or shed the virus nasally post reactivation, demonstrating its safety as a vaccine vector by eliminating the risks of productive replication and viral circulation among pigs, thus preventing reversion to virulence. The experimental design considered postmitotic neurons not coding for TK, allowing TK minus viruses to replicate in nonneuronal cells. This PRV triple mutant is highly attenuated for pigs, is immunogenic, accommodates large inserts, expresses chimeric proteins, establishes latency without replication in TG neurons, and is suitable for polyvalent vaccine development.

## 8. Strategies for Alphaherpesvirus Vaccine Development

The development of vaccines for alphaherpesviruses involves several key strategies, each aimed at inducing robust and protective immune responses while minimizing the risk of reactivation or disease (Table 1). Live-attenuated vaccines use a weakened form of the virus that can still replicate but is unable to cause disease. They elicit strong, long-lasting immunity by closely mimicking natural infection [87]. Examples include the PRV Bartha-K61 strain and BoHV-1 gE-deleted vaccines, both of which have been widely used in veterinary medicine. The identification of virulence-associated genes, particularly those related to neurovirulence, is a key step in the construction of live-attenuated vaccines for alphaherpesviruses. Based on the current research progress surrounding latency and reactivation genes, we list the potential genes that can be knocked out and their functions in Table 2. The study of these genes provides new strategies and approaches for the development of live-attenuated vaccines for alphaherpesviruses. In addition, inactivated vaccines contain viruses that are no longer able to replicate. They are safer than live vaccines but often require adjuvants or multiple doses to generate sufficient immunity. These vaccines have been used for PRV and BoHV-1 in livestock but may offer less durable immunity compared to live-attenuated vaccines. Subunit vaccines use specific viral proteins, such as glycoproteins, to stimulate an immune response without the risks associated with whole viruses. Glycoprotein D (gD) is a common target for HSV or PRV vaccines, and similar approaches are being explored for other alphaherpesviruses. DNA vaccines introduce plasmids encoding viral antigens directly into host cells, which then produce viral proteins and trigger immune responses. While still largely experimental for alphaherpesviruses, they offer a promising avenue due to their stability and ease of production. Viral vector vaccines, such as adenoviruses, are engineered to express alphaherpesvirus proteins. These vectors can stimulate immunity without the risk of causing disease from the target virus. Viral vector vaccines are being explored for HSV and offer a novel approach to alphaherpesvirus vaccine development. Each strategy has advantages and challenges, and ongoing research is aiming to refine these approaches to improve the efficacy and safety of these vaccines and the longevity of their protection (Table 1).

## 9. Conclusions and Future Prospects

In conclusion, an in-depth understanding of the latency and reactivation mechanisms behind alphaherpesviruses provides valuable guidance for designing attenuated alphaherpesvirus vaccines. In future vaccine designs, it is essential not only to knock out the main virulence factors but also to consider genes related to viral latency and the transport of viral particles within neurons. This will enhance vaccine safety by preventing latent infection or reactivation within neural cells. Here, we have summarized the relevant genes that potentially impact the safety of attenuated alphaherpesvirus vaccines (neuronal transport-related genes and latency- and reactivation-related genes) (Table 1). Targeted mutation or deletion of these key viral genes can improve vaccine efficacy and safety, aiding in the control and prevention of recurrent infections caused by these viruses. Research on the genes related to alphaherpesvirus latency and reactivation has significantly advanced our understanding of viral pathogenesis and host–virus interactions. Continued investigation into the molecular mechanisms governing latency establishment, maintenance, and reactivation will pave the way for the development of effective therapeutics and vaccines against alphaherpesvirus infections.

## Figures and Tables

**Table 1:** The main strategies for alphaherpesvirus vaccine development and the advantages and disadvantages of each strategy.

Strategy	Advantages	Disadvantages
Live-attenuated vaccines	Strong, long-lasting immune response. Mimics natural infection.	Risk of reversion to virulence and reactivation. May not be safe for immunocompromised individuals.
Inactivated vaccines	Safe, no risk of causing disease. Stable for storage and transport.	Weaker immune response. Often requires multiple doses or adjuvants.
Subunit vaccines	Highly specific and safe. No risk of infection.	Weaker immune response. May require adjuvants or boosters.
DNA vaccines	Stable and easy to produce. Induces both humoral and cellular immunity.	Still experimental for alphaherpesviruses. May require improved delivery methods.
Viral vector vaccines	Can induce strong immunity. No risk from the target virus.	Potential for immune response against the vector. Safety concerns in certain populations.

**Table 2:** Neuronal transport-related, latency-related, and reactivation-related genes that potentially impact the safety of attenuated alphaherpesvirus vaccines.

Viral Gene(s)	Virus	Proposed Function(s)	Reference
TK	HSV-1/HSV-2/BHV-1/PRV	The thymidine kinase (TK) enzyme is an important viral product involved in nucleotide synthesis, making the TK gene a common target for herpesvirus attenuation. Crucial for latent PRV reactivation and necessary for HSV-1 reactivation, but unnecessary for latency establishment.	[75,76,78,79,80,81,82]
gE/gI	HSV-1/PRV/BHV-1	Important for efficient virus latency establishment and reactivation in neurons due to its capacity for promoting virus neuroinvasion. The gE/gI complex is not essential for transneuronal spread in vivo. It is a common target for herpesvirus attenuation.	[50,51,56,57,58,59,60]
US9	HSV-1/PRV	US9 membrane protein controls the axonal localization of diverse viral membrane proteins but not that of capsid or tegument proteins. Affects virus reactivation due to its critical role in promoting virus anterograde transport in neurons.	[46,47,48,50,52,53,55,61]
gK	HSV-1/HSV-2/other primate viruses belonging to the Simplexvirus genus	US11 is an immune antagonist produced by both HSV-1 and HSV-2, as well as other primate viruses belonging to the Simplexvirus genus. US11 plays a significant role in disease progress and reactivation from latency. HSV-1 gK is essential for efficient replication and spread in the corneal epithelium and trigeminal ganglia neuroinvasion.	[74,83]
UL36	HSV-1/other Herpesviridae	Homologs of the essential large tegument protein UL36 of herpes simplex virus 1 are conserved throughout the Herpesviridae complex with pUL37 and form part of the capsid-associated “inner” tegument. UL36 is crucial for transport of the incoming capsid and docking at the nuclear pore early after infection as well as for virion maturation in the cytoplasm. The dynamic ubiquitination of the PRV UL36 protein significantly affects the virus’s ability to invade neurons. UL36 recruits molecular motors to facilitate retrograde transport.	[66,67,68]
UL37	HSV-1/PRV/BHV-1	A capsid-bound tegument protein, UL37 is an essential effector of retrograde axonal transport and also houses a deamidase activity that antagonizes innate immune signaling. The pUL37 tegument protein has a surface region that is an essential neuroinvasion effector. These proteins support long-distance retrograde axonal transport and invasion of the nervous system in vivo.	[29,70,71]
UL21	PRV	The PRV UL21 gene is a major determinant of PrV virulence and point mutations affecting the UL21 gene of live vaccine strain Bartha contribute to its attenuated phenotype. UL21 affects the kinetics of retrograde transneuronal infection in vitro and in vivo.	[72,73]
